# Biochemical characterization of extremozyme L-asparaginase from *Pseudomonas* sp. PCH199 for therapeutics

**DOI:** 10.1186/s13568-023-01521-2

**Published:** 2023-02-24

**Authors:** Sanyukta Darnal, Vijeta Patial, Virender Kumar, Subhash Kumar, Vijay Kumar, Yogendra S. Padwad, Dharam Singh

**Affiliations:** 1 Molecular and Microbial Genetics Lab, Biotechnology Division, CSIR-Institute of Himalayan Bioresource Technology, Palampur, Himachal Pradesh 176 061 India; 2 Academy of Scientific and Innovative Research (AcSIR), Ghaziabad, 201 002 India; 3 Dietetics & Nutrition Technology Division, CSIR-Institute of Himalayan Bioresource Technology, Palampur, Himachal Pradesh 176 061 India

**Keywords:** Periplasmic L-asparaginase, Osmotic shock method, Protein purification, Cytotoxicity, Himalayan niche

## Abstract

L-asparaginase (L-ASNase) from microbial sources is a commercially vital enzyme to treat acute lymphoblastic leukemia. However, the side effects associated with the commercial formulations of L-ASNases intrigued to explore for efficient and desired pharmacological enzymatic features. Here, we report the biochemical and cytotoxic evaluation of periplasmic L-ASNase of *Pseudomonas* sp. PCH199 isolated from the soil of *Betula utilis*, the Himalayan birch. L-ASNase production from wild-type PCH199 was enhanced by 2.2-fold using the Response Surface Methodology (RSM). Increased production of periplasmic L-ASNase was obtained using an optimized osmotic shock method followed by its purification. The purified L-ASNase was a monomer of 37.0 kDa with optimum activity at pH 8.5 and 60 ℃. It also showed thermostability retaining 100.0% (200 min) and 90.0% (70 min) of the activity at 37 and 50 ℃, respectively. The *K*_m_ and *V*_max_ values of the purified enzyme were 0.164 ± 0.009 mM and 54.78 ± 0.4 U/mg, respectively. L-ASNase was cytotoxic to the K562 blood cancer cell line (IC_50_ value 0.309 U/mL) within 24 h resulting in apoptotic nuclear morphological changes as examined by DAPI staining. Therefore, the dynamic functionality in a wide range of pH and temperature and stability of PCH199 L-ASNase at 37 ℃ with cytotoxic potential proves to be pharmaceutically important for therapeutic application.

**Supplementary Information:**

The online version contains supplementary material available at 10.1186/s13568-023-01521-2.

## Introduction

The enzymes that deprive nutrients for neoplastic cells present a promising approach for treating malignancies, and L-ASNase occupies the center stage of it (Husain et al. 2016). L-ASNase (E.C. 3.5.1.1) is a commercially important enzyme due to its application in acute lymphoblastic leukemia (ALL) treatment. It is a well established fact now that most leukemic cells rely on an exogenous supply of L-asparagine for survival. The antileukemic impact of L-ASNase is due to the quick exhaustion of the circulating L-asparagine from the blood (Batool et al. 2015). The reduced level of L-asparagine causes a reduction in protein synthesis, and suppression of DNA and RNA synthesis, which leads to cellular dysfunction and eventually cell death (Narta et al. 2007; Kumar et al. 2010). In contrast, normal cells can manufacture L-asparagine and are thus less affected by L-asparagine depletion during the treatment.

Commercial formulations for ALL treatment include L-ASNase from *E. coli* (Elspar^®^, Medac, Paronal^®^, and Kidrolase^®^), its pegylated form and *Dickeya dadandii* (Samson et al. 2005) L-ASNase (Erwinase^®^) (van den Berg 2011). However, the exclusive use of these L-ASNase as a treatment for ALL are associated with various side effects during the treatment. Common side effects include acute pancreatitis (Raja et al. 2012), myocardial infarction, acute hepatic dysfunctions, thrombosis, hypersensitivity reactions, anaphylaxis, and clotting disorders (Homans et al. 1987). This lays the foundation for exploring novel L-ASNases with higher chemotherapeutic potential and fewer side effects.

Bacterial L-ASNase has been a fixation of researchers for the past few decades, and various efforts were made to isolate enzyme with fewer side effects. Traditional approaches of isolating and identifying L-ASNase from various sources were well investigated. It includes, *Bacillus halotolerans* OHEM18 (El-Fakharany et al. 2022), *Brevibacillus borstelensis* ML12 (Mukherjee and Bera 2022). Further, efforts were also made to improve the properties of the existing enzymes or recombinantly reproduce it. It includes *Bacillus subtilis* 168 (Feng et al. 2019), *Pseudomonas resinovorans* IGS-131 (Mihooliya et al. 2020), *Enterobacter carcerogenus* (Kolcuoğlu and Çakmak 2022), and *Pseudomonas aeruginosa* (Qeshmi et al. 2022). However, an efficient enzyme with desired characteristics is still a fairytale.

In consistence to exploration, our group has also screened various niches in the western Himalayan region for efficient L-ASNase with improved properties compared to existing L-ASNases (Kumar et al. 2019; Kumar et al. 2022a, 2022b). Notably, the Himalayan niches such as glacier surfaces, glacier streams, and mineral-rich soil harbour microbial life with remarkable adaptive properties. Extreme conditions of the Himalayas like temperature fluctuations, survival in low oxygen, tolerance to salt and pH fluctuations, low pressure, and high UV radiations bestowed the bioresources with uniqueness (Kumar et al. 2019). Hence, realizing the importance of Himalayan niches, the existing scope for finding pH and temperature stable functional enzymes are a boon to pharmaceutical and food industries. Therefore, we report the extraction, purification to homogeneity, and characterization of periplasmic L-ASNase from Himalayan *Pseudomonas* sp. PCH199. The study revealed the periplasmic L-ASNase to be highly active in a wide range of pH and temperature with a high degree of stability at 37 ℃ (100% activity even after incubating for 200 min) than previous reports. With high substrate specificity (*K*_m_ value 0.164 mM), the PCH199 L-ASNase is also cytotoxic to K562 cancer cells with a comparatively low IC_50_ value of 0.309 U/mL than the existing L-ASNases that eventually leads to apoptosis.

## Materials and methods

### Chemicals and cell lines

Di-sodium phosphate, potassium dihydrogen phosphate, sodium chloride, magnesium sulfate heptahydrate, calcium chloride dihydrate, ammonium sulfate,  glucose and Nessler's reagent were obtained from HiMedia (Mumbai, India). Bradford reagent, L-asparagine and L-glutamine were obtained from Sigma Aldrich (St. Louis, USA). Blood cancer cell line K562 and normal cell line IEC-6 were procured from National Center for Cell Science (NCCS Pune, India).

### Isolation of bacteria, qualitative screening for L-ASNase production, and identification of L-asparaginase producing bacterium

PCH199 was isolated from the forest soil of *Betula utilis*, the Himalayan birch in the Satrundi alpine zone (32°58′31′′N, 76°13′11′′E; 3368 m above sea level) in the Pangi-Chamba region of Himachal Pradesh, India. One-gram soil was inoculated in 100 mL of enriched M9 minimal medium (components in g/L; 6.0 g Na_2_HPO_4_.2H_2_O, 3.0 g KH_2_PO_4_, 0.05% (v/v) NaCl, 2.0 mM MgSO_4_.7H_2_O, 0.1 mM CaCl_2_.2H_2_O, 1.0% (w/v) L-asparagine and 0.2% (v/v) glucose) and was incubated at 28 ℃ up to 72 h. The bacterial isolation was performed on enriched M9 medium using serial dilution as described earlier (Kumar et al. 2019). The glycerol stocks of purified colonies were prepared and stored at − 80 ℃. A previously described and modified plate assay method for qualitative screening of L-ASNase production was used (Gulati et al. 1997; Kumar et al. 2019) where M9 medium supplemented with 0.003% (v/v) phenol red (1.0 mg/mL stock prepared in ethanol) was inoculated and incubated at 28 ℃ for 24 h. The 16S rDNA sequencing-based identification was performed, and the nucleotide sequence was utilized for similarity search using NCBI BLAST search tool and the EzBioCloud server database (https://www.ezbiocloud.net/).

### Quantitative estimation of L-ASNase activity

L-ASNase activity was measured spectrophotometrically at 480 nm using Nessler’s reagent, which measures the amount of ammonia released in the reaction mixture (Imada et al. 1973). Briefly, the L-ASNase assay was carried out in a 1.0 mL volume of a reaction containing 0.45 mL Tris–HCl (50 mM, pH 8.5), 0.5 mL L-asparagine (10 mM prepared in buffer), and 0.05 mL crude L-ASNase. The reaction was incubated at 37 ℃ for 15 min and terminated by adding 0.25 mL of 1.5 M trichloroacetic acid (TCA). For the control reaction, the enzyme was added after adding TCA. The reaction was diluted as per necessity before adding Nessler’s reagent, and the absorbance was measured at 480 nm. The specific activity of purified L-ASNase was measured in U/mg protein (micromoles/min/mg). One unit (U) of L-ASNase is the amount of enzyme needed to liberate 1.0 µmol of ammonia from L-asparagine per min under standard reaction conditions. The protein content in the supernatant was measured using the Bradford reagent (Bradford 1976) with bovine serum albumin as the standard.

### Statistical optimization of L-ASNase production at flask scale using Response Surface Methodology (RSM)

The bacterial isolate PCH199 was grown at different temperatures (4, 15, 20, 28, and 37 ℃) and the culture was collected every 4 h beyond 20 h of growth until the culture reached the stationary phase. The cell growth and L-ASNase activity in extracellular, periplasmic, and intracellular environments were analyzed. The temperature (15 ℃) corresponding to the highest enzyme activity was further selected for the statistical optimization to obtain maximum enzyme production by applying Central Composite Design (CCD) in the RSM. Similarly, M9 minimal medium with sodium phosphate of various pH from 5.8–7.5 was prepared. The bacterium PCH199 was grown in the prepared media and cultured at 15 ℃. The cell growth and L-ASNase activity were observed. Stat-Ease Design-Expert Trial version 11 (Stat-Ease Corporation, Minnesota, USA) was employed to design optimization experiments. Three components of M9 medium i.e., Na_2_HPO_4_, KH_2_PO_4_ buffer molarity (0–100 mM), L-asparagine (0–2.0%), and glucose (0–0.5%), that affected the growth and enzyme production were taken into consideration for the experiment (Additional file 1: Table S1). Twenty experimental runs were carried out based on the CCD scheme, and response were evaluated using three-dimensional plots. The L-ASNase production was carried out using an optimized medium in further experiments. The final equation (in terms of coded variables) used for the optimization experiment is as follows:

1 $${\text{L}}\, - {\text{asparaginase}}\,{\text{activity}}\,\left( {{\raise0.7ex\hbox{${\text{U}}$} \!\mathord{\left/ {\vphantom {{\text{U}} {{\text{mL}}}}}\right.\kern-0pt} \!\lower0.7ex\hbox{${{\text{mL}}}$}}} \right)\, = \,0.4525\, + \,0.1411{\text{A}}\, + \,0.1678{\text{b}}\, + \,0.0226{\text{C}}\, + \,0.1610{\text{Ab}}\, + \,0.0245{\text{AC}}\, + \,0.0094{\text{bC}}\, - \,0.0614{\text{A}}^{2} \, - \,0.0547{\text{b}}^{2} \, - \,0.0646{\text{C}}^{2}$$

where A denotes buffer concentration (mM), b denotes L-asparagine concentration (%, w/v) and C denotes glucose concentration (%, v/v).

### Extraction of periplasmic L-ASNase

The production of periplasmic L-ASNase was carried out in an optimized M9 minimal medium. Overnight grown seed culture prepared in M9 medium was inoculated (2.0%, v/v) to M9 production medium. The culture was incubated at 15 ℃ in an incubator shaker at 160 RPM agitation for 28 h and harvested by centrifuging at 8000 *g* for 15 min. The bacterial pellet was thoroughly washed with 50.0 mM Tris-HCl buffer (pH 8.5), followed by extraction of the periplasmic enzyme. The extraction process was performed at 4 ℃. The osmotic shock method previously described (Neu and Heppel 1965) was used for enzyme extraction from the exponentially growing culture. Bacterial culture of cell OD equivalent to 5.0 was suspended in a hyperosmotic solution of 50.0 mM Tris–HCl (pH 8.5), 20.0% sucrose, and 0.5 mM EDTA. This solution was incubated in ice for 15 min followed by centrifugation at 12,000 *g* for 20 min. The pellet obtained was resuspended in ice-cold distilled water and kept on ice for 15 min and then centrifuged at 12,000 *g* for 20 min. The supernatant containing the extracted periplasmic L-ASNase was used as a cell-free extract or crude enzyme fraction.

### Purification of periplasmic L-ASNase

The enzyme was purified in 3 steps. The crude was subjected to ammonium sulfate precipitation, anion exchange, and size exclusion chromatography to achieve homogenous purification. All the purification steps were performed at 4 ℃.

#### Ammonium sulfate precipitation of periplasmic extract

The crude periplasmic fraction extracted was subjected to protein estimation and activity analysis. Periplasmic L-ASNase was concentrated using the ammonium sulfate precipitation method, where finely powdered ammonium sulfate was added to the crude to achieve 80.0% saturation with overnight precipitation at 4 ℃ in stirring conditions. The precipitated protein was collected by centrifugation at 12,000 *g* for 15 min and further dissolved in 25 mM Tris–HCl buffer (pH 8.2). The protein sample was dialyzed at 4 ℃ using a dialysis membrane (14 kDa) at 4 ℃ against the same buffer by changing the buffer three times after 4 h interval.

#### Anion exchange chromatography

Q-Sepharose (GE Healthcare, Chicago, USA) column was prepared and equilibrated with Tris-HCl buffer (25 mM Tris–HCl, pH 8.2). The dialyzed protein was filtered using a 0.45 µm filter and was loaded onto the column. The protein was eluted with 1.0 M NaCl using a stepwise gradient (0–1.0 M) with a flow rate of 2.0 mL/min. The active fractions were collected, dialyzed, and concentrated using Amicon centrifugal filters (30 kDa MWCO, Merck Millipore, Massachusetts, USA).

#### Size exclusion chromatography

The concentrated protein fraction was loaded onto the Superdex 200 column (GE Healthcare, Chicago, USA) pre-equilibrated with 25 mM Tris-HCl (pH 8.2). The protein elution was done using the same buffer at a flow rate of 0.5 mL/min. The protein fractions were analyzed for enzyme activity, and protein was estimated for each fraction. The protein purity and molecular weight was determined by fractionating protein on SDS-PAGE (Laemmli 1970).

### Biochemical characteristics of purified L-ASNase

#### Effect of pH and temperature on the activity of the L-ASNase

The following 50.0 mM concentration buffers with different pH were prepared; citrate (pH 4.0–6.0), sodium phosphate (6.0–8.0), Tris–HCl (8.0–10.0), carbonate bicarbonate (9.0–10.0), potassium chloride NaOH (10–12), and bicarbonate NaOH (12.0–13.0). The optimum pH was determined by evaluating the enzyme activity at different buffer pHs. The optimal temperature for L-ASNase activity of the purified enzyme was analyzed by performing the enzyme assay in a temperature range of 10–75 ℃. Finally, the enzyme activity was expressed in terms of relative activity.

#### Thermostability

The purified enzyme was incubated at temperatures ranging from 37–80 ℃ to check its thermostability. Residual enzyme activity was analyzed at different time intervals until enzyme’s activity decreased to half than initial enzyme activity. The enzyme activity was expressed as residual activity.

#### Effect of metal ions and protein modifying agents

The effects of various metal ions (Cu^2+^, Co^2+^, K^+^, Na^+^, Ca^2+^, and Zn^2+^), and protein modifying agents [sodium dodecyl sulfate (SDS), dimethyl sulfoxide (DMSO), ethylenediaminetetraacetic acid (EDTA), and phenylmethylsulfonyl fluoride (PMSF)] on purified L-ASNase activity were determined. The enzyme was pre-incubated with individual metal ions and protein modifying agent’s solutions in a 1.0 mM concentration for 60 min at 37 ℃, followed by an estimation of enzyme activity. The residual activity was measured under the standard enzyme assay conditions and the activity was conveyed in terms of relative activity.

#### Kinetic parameters of purified L-ASNase

The enzyme activity of purified L-ASNase was assayed using different L-asparagine concentrations (0.1–3.0 mM). Non-linear regression analysis was done and Michaelis-Menten constant (*K*_m_) and maximum velocity (*V*_max_) of the purified enzyme were calculated by plotting using Graphpad prism software (version 8). The other kinetic parameter (*k*_cat_*,* and *k*_cat_*/K*_m_) were determined using the Michaelis–Menten equation where *k*_cat_ = *V*_max_ / [E_0_], [E_0_] is the initial enzyme concentration and *V*_max_ is the maximum reaction rate (μmole/min) in the assay*.*

#### Cytotoxicity evaluation of purified L-ASNase against K562 blood cancer cells

Cytotoxic activity of the purified L-ASNase was evaluated using MTT [3-(4, 5-dimethylthiazol-2-yl)-2,5-diphenyl tetrazolium bromide] assay (Mosmann 1983). Blood cancer cell line K562 and normal cell line IEC-6. K562 cell line was procured from NCCS, Pune, India. Cells were seeded in a 96 well plate at a density of 10 × 10^4^ cells per well. Cells were treated with varying concentrations of purified L-ASNase and commercial *E. coli* L-ASNase (Elspar), and incubated at 37 ℃ in 5.0% CO_2_ for 24 and 48 h. After incubation, cells were treated with 10.0% MTT (5.0 mg/mL) dye overnight for the formation of formazan crystals. The crystals were dissolved using dimethyl sulfoxide (DMSO) and the cell cytotoxicity was determined at 570 nm on a microplate reader.

#### Nuclear morphology evaluation using DAPI stain and fluorescent microscopy

Changes in the cells' nuclear morphology after treatment were evaluated by staining the cells with 4′,6-diamidino-2-phenylindole dihydrochloride (DAPI) stain. Cells were seeded in a 96-well plate at a density of 15 × 10^4^ cells per well with IC_50_ value of purified L-ASNase from PCH199 strain and incubated at 37 ℃ in 5.0% CO_2_ for 24 h. Further, treated cells were fixed using paraformaldehyde (4.0%) for 30 min. Phosphate buffer saline (PBS) was used to wash the cells, and DAPI stain (1.0 µg/mL) was used to stain them for 15 min in the dark. Fluorescence microscopy was used to examine the stained cells (Mazloum-Ravasan et al. 2021).

## Results

### Isolation, qualitative and quantitative screening, and identification of L-ASNase producing bacterium

The bacterium PCH199 was isolated on an enriched M9 minimal medium supplemented with L-asparagine (1.0%, w/v) and glucose (0.2%, v/v). Qualitative analysis performed on M9 medium supplemented with phenol red as an indicator produced a clear zone of color change from yellow to pink after 24 h of incubation at 28 ℃, indicating L-ASNase production (Additional file 1: Fig. S1). 16S rDNA sequencing analysis revealed 99.06% sequence similarity of PCH199 to *Pseudomonas glycinae* MS586(T), Accession number ON782287 (Additional file 1: Fig. S2). Quantitative estimation using the cell-free extracellular fraction revealed 0.68 U/mL L-ASNase activity with undetectable L-GLNase activity at 36 h of production. However, a variable extracellular L-ASNase activity was noticed from batch-to-batch run. Further to overcome the variation in enzyme activity, periplasmic protein extraction was performed. Both L-ASNase and L-GLNase activity of 0.34 and 0.31 U/mL, respectively, from crude periplasmic fraction were obtained after extraction.

### Statistical optimization of L-ASNase production at flask scale using Response Surface Methodology (RSM)

**Fig. 1:**
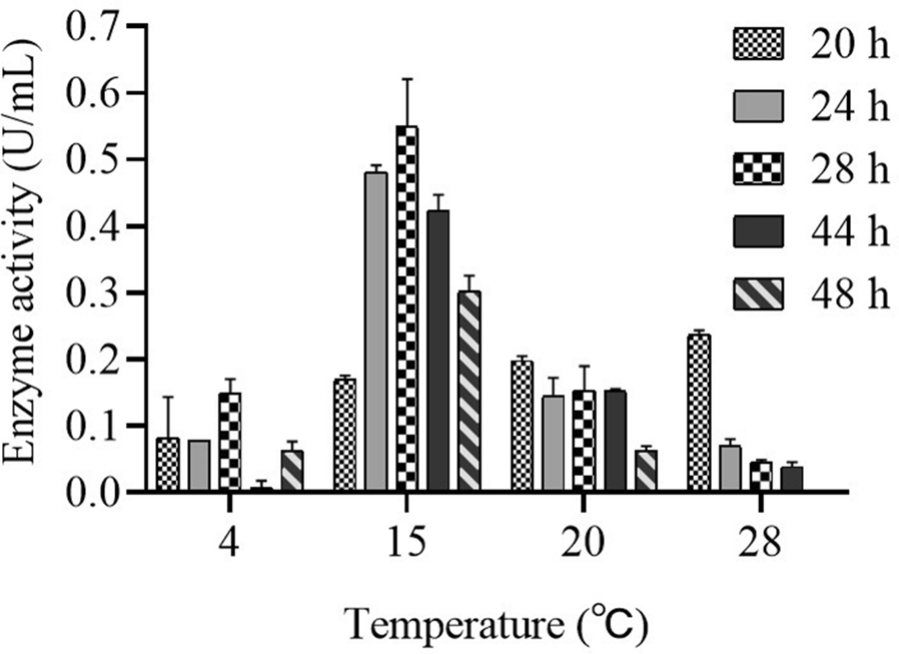
Graphical representation of periplasmic L-ASNase activity when PCH199 was cultured at different temperatures (4, 15, 20, and 28 ℃). PCH199 was grown at different temperatures and periplasmic L-ASNase activity was measured at different time intervals from the culture of PCH199 corresponding to each temperature

PCH199 was incubated at different temperatures (4, 15, 20, 28, and 37 ℃), and the maximum L-ASNase activity of 0.650 U/mL crude was observed at 15 ℃ after 28 h (Fig. 1). The cell growth was measurable in all temperatures except 37 ℃. No substantial difference in the periplasmic L-ASNase activity was observed when the bacterium PCH199 was grown in M9 medium with different pH (Additional file 1: Fig. S4). The results of the response surface model, including observed response values, are given in Additional file 1: Table S1. It was found that the presence of L-asparagine in the medium is highly necessary for L-ASNase production, indicating the auxotrophic growth requirement. None of the enzyme activity was observed without L-asparagine in the medium (Run 17, Additional file 1: Table S1). Similarly, buffer concentration was also found to be critically important, and no activity was observed even if the medium was supplemented with L-asparagine and glucose (Run 5, Additional file 1: Table S1). The absence of glucose did not drastically affect the enzyme activity (Run 6 and 9, Additional file 1: Table S1). The analysis of the variance (ANOVA) on the data obtained after performing the RSM experimental design is given in Additional file 1: Table S1. The *p-*value was 0.0394, suggesting that the experimental model was significant in enhancing the L-ASNase production. Moreover, the R^2^ value (correlation coefficient) was 0.95 proposing a good regression fit of the experimental model. Additionally, the R^2^ adjusted value showed the correction decision coefficient was 0.89. The results and response of the model suggested a good fit model (Additional file 1: Table S2).

**Fig. 2:**
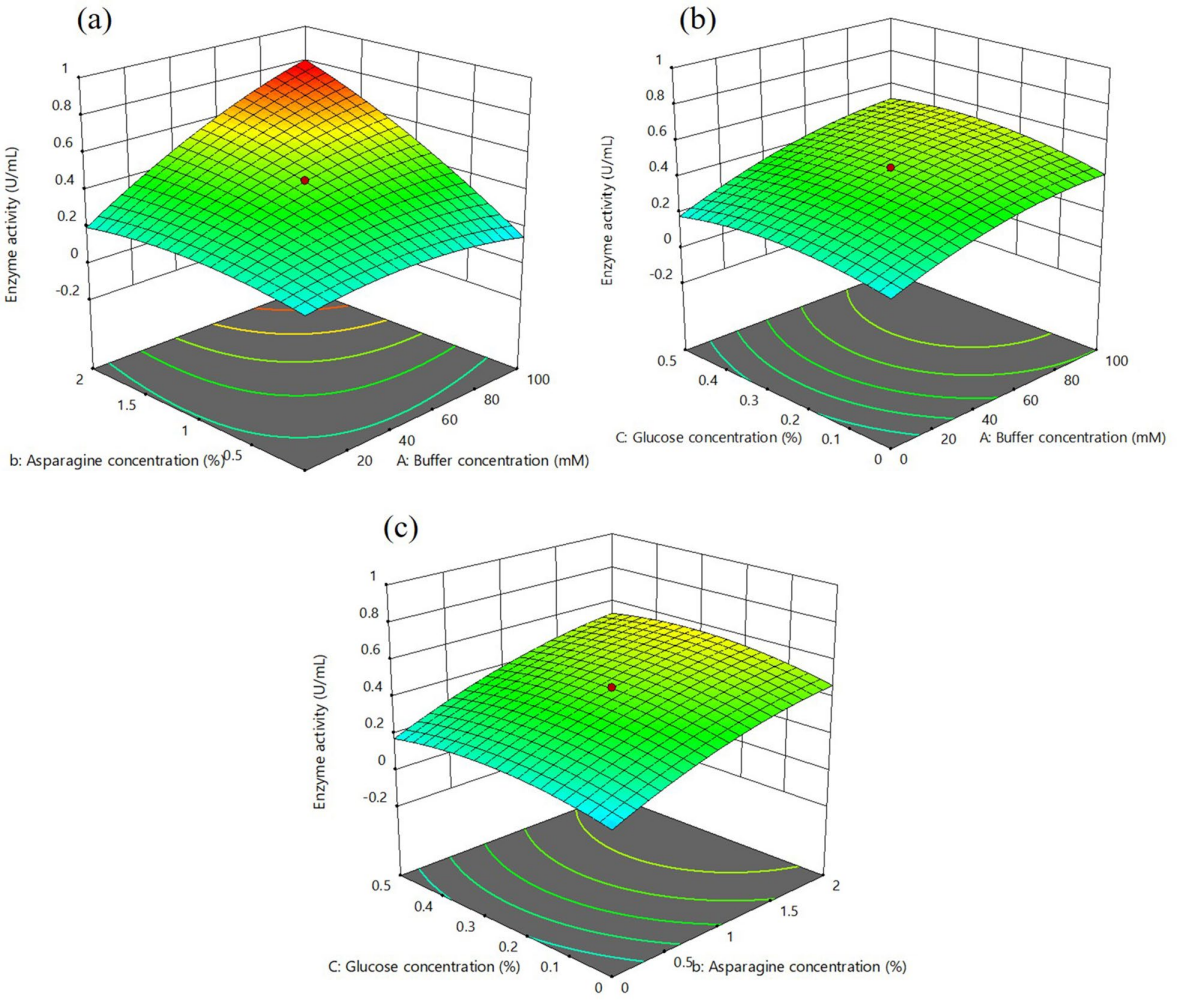
Statistical optimization of production medium using RSM. Three-dimensional response surface plots showing the interactive effects of selective variable on L-ASNase activity, **a** L-asparagine (%, w/v) and buffer concentration (mM), **b** glucose (%, v/v) and buffer concentration (mM) and **c** L-asparagine (%, w/v), and glucose concentration (%, v/v)

 CCD showed the positive impact of buffer and L-asparagine concentration, leading to increased enzyme activity compared to glucose concentration. Three-dimensional response surface graphs (Fig. 2a–c) were generated to recognize the relationship of individual medium components to each other. The buffer concentration and L-asparagine concentration had strong interaction between them for L-ASNase activity (*p*-value for the interaction term Ab = 0.0003) (Additional file 1: Table S2). Glucose had negligible contribution to enhance enzyme activity apart from supporting growth (*p*-value for the interaction AC = 0.3, bC = 0.7) (Additional file 1: Table S2). The highest activity of 0.76 U/mL was obtained with a medium combination (buffer 100 mM, asparagine 2.0% and glucose 0.5%), which is close to the predicted value of 0.79 U/mL. The model validation was performed to analyze its significance for enhancing L-ASNase activity. The L-ASNase activity obtained during the model confirmation was 0.78, which is close to the predicted value of 0.79. The statistical optimization led to a 2.2-fold increase in the enzyme activity compared to the unoptimized medium. However, no further increase in the enzyme activity was observed when the L-asparagine and buffer concentrations were increased beyond 2.0% and 100 mM, respectively. The maximum activity was obtained within parameters given by the RSM and were in the design region. This suggested the significance of the given model in enhancing the L-ASNase production under optimized conditions.

### Extraction and purification of periplasmic L-ASNase

**Fig. 3:**
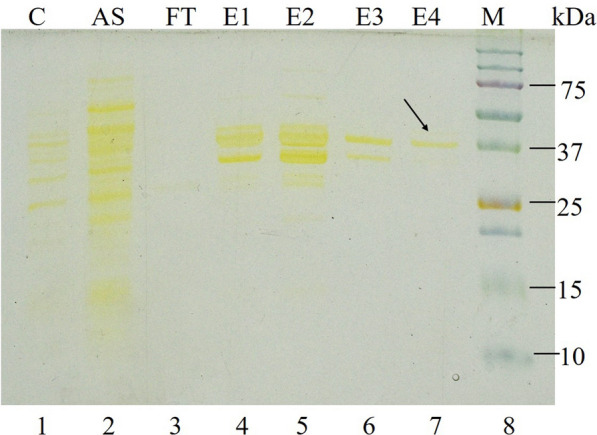
SDS-PAGE analysis of purified PCH199 L-ASNase. Proteins were separated on 10% SDS-PAGE and stained with silver stain. Lane 1 (C), cell-free periplasmic extract; Lane 2 (AS), ammonium sulfate precipitation extract; Lane 3 (FT), flow-through; Lane 4 and 5 (E1, E2), eluted fractions of anion-exchange chromatography; Lane 6 and 7 (E3, E4), eluted fractions of size-exclusion chromatography; Lane 8 (M), protein molecular weight marker (kDa). Arrow indicates homogenous purified protein

The extracted periplasmic L-ASNase with 0.76 U/mL crude activity from the optimized medium was purified to homogeneity and the sequential purification is summarized in Table 1. The maximum periplasmic L-ASNase activity was recovered in 80% ammonium sulfate precipitation. The dialyzed L-ASNase had a specific activity of 1.35 U/mg with 2.7-fold purification. In anion-exchange chromatography, the purification fold was 76.68 with a specific activity of 38.34 ± 2.12 U/mg. Finally, with size-exclusion chromatography, a total of 0.294 mg L-ASNase with 4.15% yield was achieved with L-ASNase activity being 47.18 ± 0.42 U/mg activity. SDS-PAGE analysis was performed to evaluate the purified protein from sequential purification steps (Fig. 3). A combination of anion-exchange and size-exclusion chromatography revealed a single distinct band of molecular weight of 37.0 kDa in SDS-PAGE analysis (Fig. 3).

**Table 1:** Summary of sequential purification of periplasmic L-ASNase from *Pseudomonas* sp. PCH199

Purification steps	Volume (mL)	Protein mg/mL	Total protein (mg)	Specific activity (U/mg)	Total activity (U)	Yield (%)	Purification fold
Crude extract	810	0.825	668.25	0.5	334.125	100	1
(NH_4_)_2_SO_4_ precipitation	90	1.725	155.25	1.35	209.588	62.727	2.7
Anion exchange chromatography	2	1.5	3	38.34	115.02	34.424	76.68
Size exclusion chromatography	2	0.147	0.294	47.18	13.871	4.151	94.36

### Effect of pH and temperature on L-ASNase activity

**Fig. 4:**
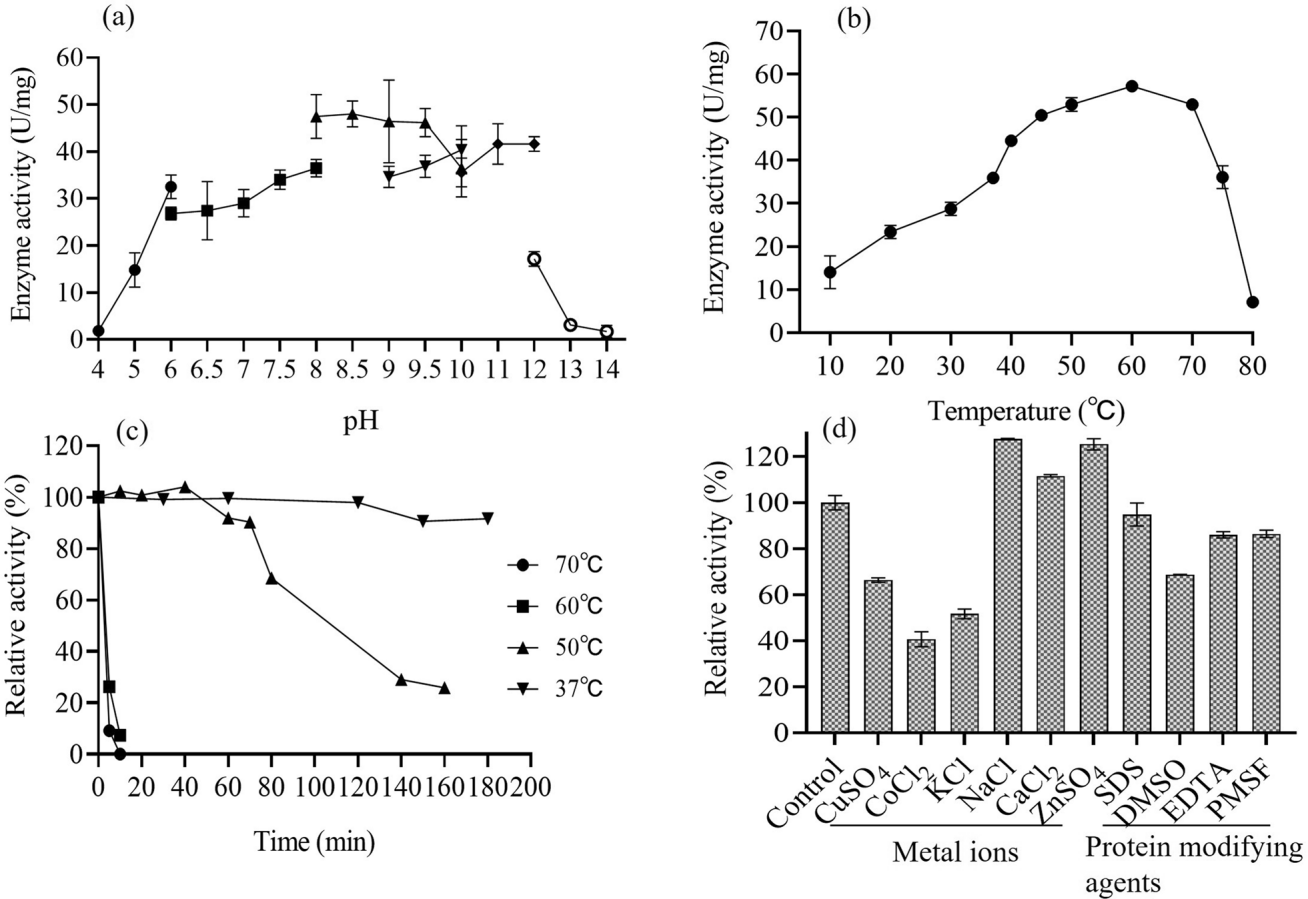
Effect of different physicochemical parameters on PCH199 L-ASNase activity. Effect of **a** different buffer pH, **b** incubation temperature, **c** thermal stability at different temperatures (37, 50, 60, and 70 ℃) and **d** metal ions and protein modifying agents (1.0 mM) on purified PCH199 L-ASNase activity

The purified enzyme from PCH199 was found to be active in a wide range of buffer pH (50 mM). The optimum activity of 48.05 U/mg in 50.0 mM Tris-HCl buffer (pH 8.5) was observed, followed by 47.48 and 46.43 U/mg activity at 8.0 and 9.0 pH, respectively (Fig. 4a). It was observed that the enzyme activity decreased at extreme acidic (pH 4.0) and alkaline conditions (pH 13.0). Likewise, the enzyme was active at a wide temperature range with a maximum activity of 57.19 U/mg recorded at 60 ℃ followed by 52.92 and 52.93 U/mg at 70 and 50 ℃, respectively. However, the enzyme activity of 35.92 U/mg for L-ASNase was obtained at 37 ℃. It was noted that the enzyme retains its activity till 70 ℃, beyond which the activity decreases considerably (Fig. 4b). Evaluation of the stability of purified L-ASNase at different temperatures revealed that it retained 100% of its activity after 200 min of incubation at 37 ℃. 90% of enzyme activity was present for 70 min when incubated at 50 ℃ (Fig. 4c). The enzyme lost 90% of its activity at 5 and 10 min when incubated at 70 and 60 ℃, respectively.

### Effect of metal ions, inhibitors, and surfactants on L-ASNase activity

The enzyme activity decreased to 66.3, 40.6, and 51.6% by Cu^2+^, Co^2+^, and K^+^, respectively. The enhanced enzyme activity of 127.5, 111.6, and 125.2% was observed in the presence of Na^+^, Ca^2+^, and Zn^2+^, respectively (Fig. 4d). Enzyme activity was not affected by 1.0 mM of protein modifying agents such as SDS, PMSF, and EDTA, while the addition of DMSO showed a 68.69% relative activity (Fig. 4d).

### Kinetic parameters of purified L-ASNase

**Fig. 5:**
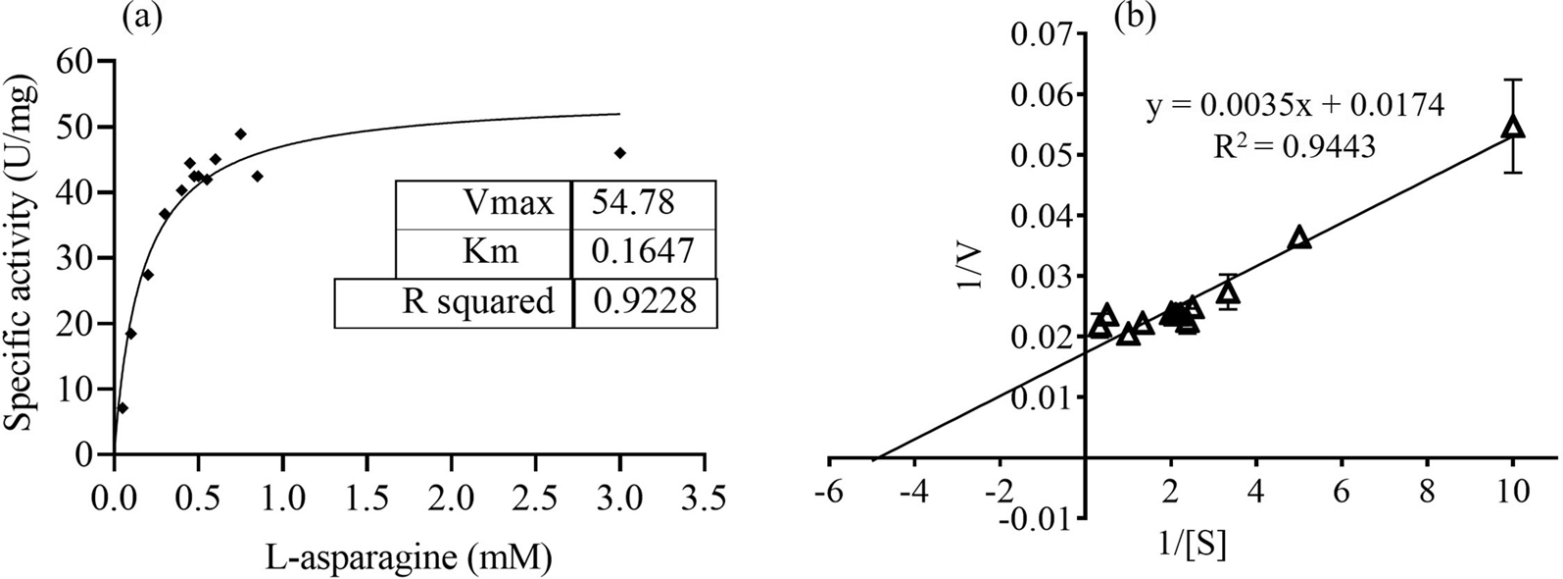
Graphical representation of kinetic study of PCH199 L-ASNase. Determination of *K*_m_ and *V*_max_ of purified L-ASNase for L-asparagine by non-linear regression analysis of experimental steady-state data. **a** Plot of the reaction velocities (V) versus substrate concentration (S: 0.1– 3.0 mM) fitted to the Michaelis–Menten equation. **b** The corresponding Lineweaver–Burk plot (*K*_m_ = 0.164 mM and *V*_max_ = 54.78 U/mg) of L-ASNase catalyzed reaction

Enzyme kinetic study of the purified L-ASNase revealed gradual increase in activity upon increasing the L-asparagine substrate concentration from 0.1 to 3.0 mM (Fig. 5a). The higher L-asparagine concentration (3.0 mM) showed saturation for its conversion by the enzyme (Fig. 5a). The *K*_m_ and *V*_max_ values for L-asparagine calculated were 0.164 ± 0.009 mM and 54.78 ± 0.4 U/mg, respectively. The *k*_cat_ and *k*_cat_/*K*_m_ for L-asparagine was 33.78 ± 2.05 s^−1^ and 205.98 ± 22 s-^1^ mM^−1^, respectively (Fig. 5b). Similarly, *K*_m_ and *V*_max_ values for L-glutamine were also determined and was found to be 0.034 mM and 57.89 U/mg, respectively (Additional file 1: Fig. S3).

### Cytotoxicity evaluation of purified L-ASNase against K562 blood cancer cells and nuclear morphology evaluation using DAPI stain

**Fig. 6:**
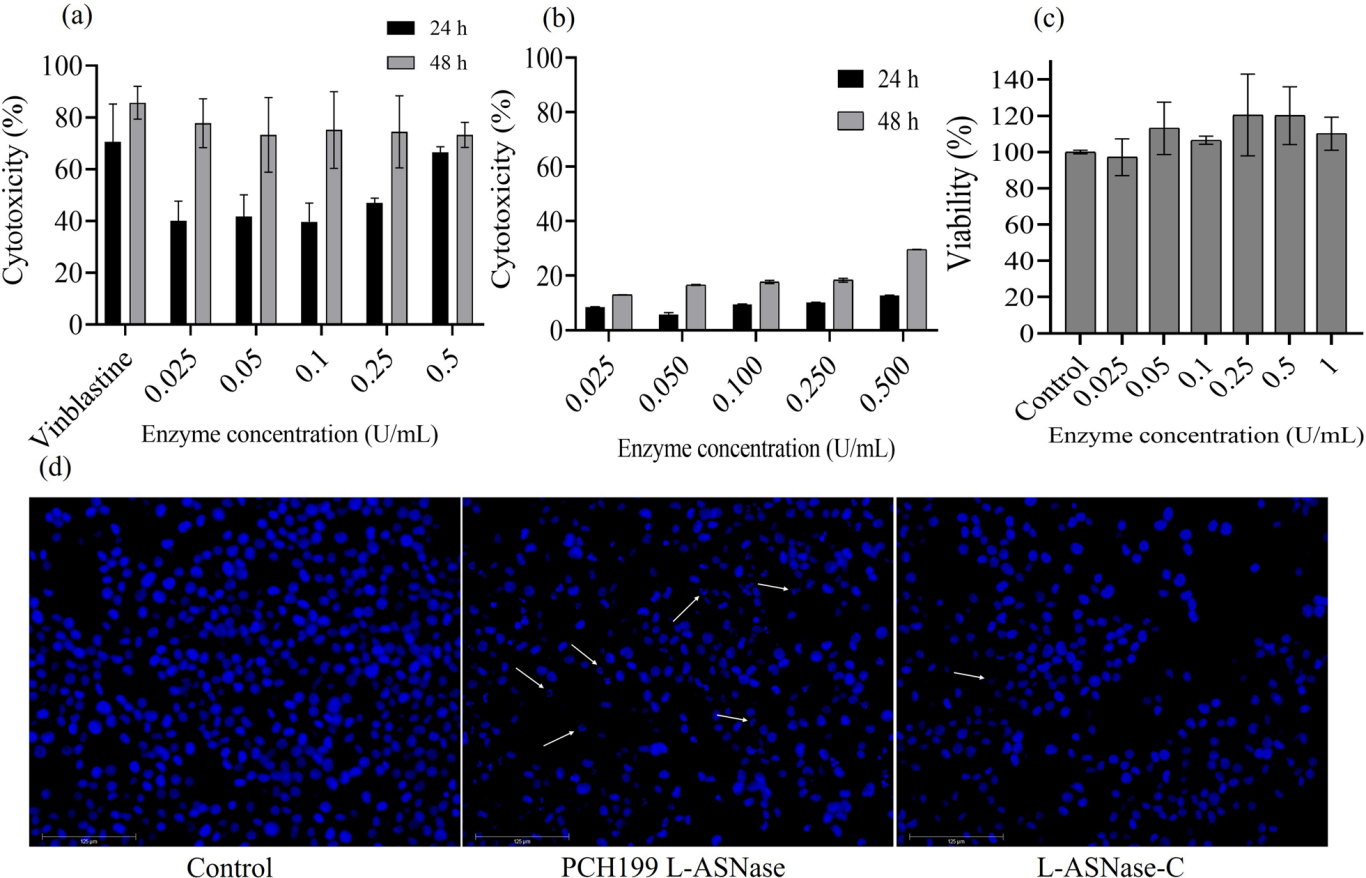
Cytotoxicity evaluation of purified L-ASNase against cancer cell lines. **a** Cytotoxic effect of purified L-ASNase from PCH199. **b** *E. coli* L-ASNase (Elspar) on K562 blood cancer cell line using MTT assay. **c** The cytotoxic effect of L-ASNase was also tested towards normal cancer cell line IEC-6. **d** DAPI staining. Treated cells stained with DAPI clearly showed DNA fragmentation. Control cells showed uniform, rounded nuclei. L-ASNase-C indicates *E. coli* L-ASNase

K562 cells efficiently responded to L-ASNase at a low concentration. MTT assay revealed that incubation of the cells with increasing concentrations of L-ASNase resulted in dose-dependent cytotoxicity. The effect of various concentrations of L-ASNase at 24 and 48 h is given in Fig. 6a. IC_50_ was calculated to be 0.309 U/mL at 24 h. A comparative dose of commercial *E. coli* L-ASNase revealed less cytotoxicity as compared to PCH199 L-ASNase (Fig. 6b). PCH199 L-ASNase was non-cytotoxic to normal cell line IEC-6 (Fig. 6c). As observed in Fig. 6d, L-ASNase treatment resulted in visible cell shrinkage, DNA fragmentation, and loss of normal nuclear architecture, all physical markers of apoptosis. However, control (untreated) cells with undamaged nuclei showed no change in the nuclear morphology.

## Discussion

L-ASNase from *E. coli,* its pegylated form, and *Dickeya dadantii* is a critical chemotherapeutic drug to treat acute lymphoblastic leukemia (ALL). The side effects associated with these drugs include hepatic dysfunctions, thrombosis, hypersensitivity, anaphylaxis, and clotting disorders. Therefore, there is a continuous surge in the discovery of bacterial L-ASNase with unique and novel properties. The western Himalayas are home to organisms that survive conditions of drastic fluctuations in physicochemical parameters. Microbes bestowed with extremozymes have shown outstanding stability that may bridge the gap between chemical and biological processes (Schiraldi and De Rosa 2002). The pharmaceutical industry can benefit from bacterial L-ASNases with excellent substrate-specificity and moderate optimum temperature (Li et al. 2019). Thus, the present study is focused on L-ASNase from the bacterial strain PCH199 isolated from the high-altitude Himalayan niche. The extremozyme has shown activity in a wide range of pH and temperatures, and various metal ions in addition to the efficient cytotoxicity against K562 cancer cells. The most important feature of the enzyme is its stability at 37 ℃, which is comparatively better than other reports in the literature so far.

The ability of PCH199 to produce L-ASNase was verified by developing a pink-colored zone around the colonies in the M9 medium supplemented with the indicator dye phenol red (Additional file 1: Fig. S1). The phenol red indicator dye is yellow in acidic conditions and turns pink at alkaline pH (Mahajan et al. 2013). The resulting color change from yellowish to pink is due to the formation of ammonia, changing medium pH after hydrolysis of asparagine. The change of medium color could be either due to extra or intracellular enzyme activity. L-ASNase is of two types, type I (cytoplasmic) and type II (periplasmic) with the latter exhibiting cytotoxic behavior towards cancer cells (Cedar and Schwartz 1967; van den Berg 2011). In the whole-genome of *Pseudomonas* sp. PCH199 there are three L-ASNase out of which two are type I and one is type II (Kumar et al. 2022b). Due to high batch-to-batch variation in extracellular activity and the presence of type II L-ASNase in the genome, we focused on the type II L-ASNase using periplasmic extraction method. Undetectable L-GLNase activity was observed in the extracellular fraction, but after periplasmic extraction L-GLNase activity was recorded. This might be due to the change in the surrounding environment that may have affected protein folding. It has been earlier reported that the osmotic shock method using Tris-HCl/sucrose/EDTA (TSE) solution is to obtain the cleanest periplasmic and cell envelope proteins, as well as to distinguish contaminants co-release (Quan et al. 2013; Costa-Silva et al. 2020).

RSM is an effective method to identify and optimize the best possible factors for increasing enzyme production. Statistical optimization of the production medium using RSM revealed that buffer and L-asparagine concentration have a positive effect on activity (Fig. 2). Previously, *Enterobacter* sp. was reported to double its enzyme activity (5.8 IU/mL) at 25 ℃ in the presence of 1.0% L-asparagine (Erva et al. 2017). Similarly, *Pseudomonas aeruginosa* SN004 increased L-ASNase production with an increased concentration of KH_2_PO_4_ in the presence of a low glucose concentration (0.2%) (Badoei-Dalfard 2015). *Pectobacterium carotovorum* MTCC 1428 exhibited 8.3-fold production of L-ASNase in a medium composed of glucose (0.20%), L-asparagine (0.52%), KH_2_PO_4_ (0.17%), and MgSO_4_.7H_2_O (0.037%) (Kumar et al. 2009). The L-asparagine in the medium acts as an inducer for L-ASNase synthesis, therefore, optimizing L-asparagine concentration may have a significant impact on the synthesis of the enzyme (Patel et al. 2022). Thus, L-asparagine is an important factor for enhanced production of L-ASNase, validated in the present study too. The solubility of the L-asparagine is low (2.94 g/100 mL). Beyond the mentioned concentration, L-asparagine does not dissolve, therefore, cannot be used as anticipated theoretically. Thus, the run that yielded the maximum activity was taken into consideration.

Purifying L-ASNase is vital to determine the anti-carcinogenic activity besides characterizing biochemical and kinetic properties for medicinal and industrial usage (Muneer et al. 2020). L-ASNase was purified to homogeneity after size exclusion chromatography (Table 1). In a similar study, *Actinomycetales bacterium* BkSoiiA L-ASNase was purified using DEAE cellulose and Sephadex G-100 with 95-fold purification and activity of 204.3 U/mg (Dash et al. 2016). *Streptomyces rochei* and *Bacillus halotolerans* OHEM18 L-ASNases were purified using ion exchange chromatography with 16.18 and 3.84-fold purification, respectively (El-Naggar and El-Shweihy 2020; El-Fakharany et al. 2022). Even though similar sequential purification steps are employed to achieve the highest level of the purified enzyme, the purification yield and fold vary with various sources, suggesting protein differences in the cuture filtrate (Meghavarnam et al. 2015).

SDS-PAGE analysis of purified enzyme revealed the monomeric molecular weight of the enzyme to be 37.0 kDa, consistent to those of commercially available L-ASNase of 37.2 kDa of *E. coli* (Kotzia and Labrou 2007) and 37.0 kDa of *Erwinia chrysanthemi* 3937 (Khushoo et al. 2004). Bacterial L-ASNases are tetramer with molecular weight of 140–150 kDa (Nunes et al. 2020). The architecture of most L-ASNases are conserved (Lubkowski and Wlodawer 2021), thus the PCH199 enzyme may be a homotetramer due to its similarity in monomeric molecular weight to *E. coli* L-ASNase. Other bacterial sources of L-ASNase also exhibit similar kind of molecular weight under the 34–37 kDa range viz., *Pseudomonas aeruginosa* (Badoei-Dalfard 2015), *Bacillus altitudinis* (Prakash et al. 2020), *Vibrio cholerae* (Radha et al. 2018), *Pseudomonas* sp. PCH44 and *Pseudomonas* sp. PCH199 (Kumar et al. 2022a, 2022b).

The optimization of various physicochemical parameters influencing purified enzyme activity is essentially required to obtain the maximum enzymatic activity. Biochemical studies of PCH199 revealed the enzyme’s extremophilic features such as wide pH and temperature functionality and stability. The maximum activity of PCH199 L-ASNase was noticed in Tris–HCl buffer at pH 8.5 (Fig. 4a), indicating the alkaline nature of the enzyme. Consistently, commercial *E. coli* L-ASNase also has an optimum alkaline pH of 7.5–8.6 (Roberts et al. 1966). Bacterial L-ASNases work best in slightly alkaline conditions as evident from various reports in the literature (reviewed by Zuo et al. 2014). Purified L-ASNase from *Bacillus aryabhattai* ITBHU02 (Singh et al. 2013), *Thermococcus zilligii* (Zuo et al. 2015), and *Bacillus amyloliquefaciens* MKSE (Yim and Kim 2019) have maximum activity in Tris-HCl buffer at pH 8.5. Alkaline pH condition favors the functionality of enzyme with L-asparagine due to a low affinity of aspartate for the enzyme active site (Stecher et al. 1999). This favors hydrolysis of the substrate L-asparagine.

Extremozymes have a biotechnological advantage due to their stability and functionality in extreme conditions. These conditions include low to high temperatures, pH, salt, and organic solvents that would have drastically affected the enzyme activity (Dumorné et al. 2017). In the present study, the purified L-ASNase showed the maximum enzyme activity at 60 ℃ (Fig. 4b), though it is isolated from the cold regions in the Himalaya. A psychrotropic yeast isolated from the Antarctic region has also shown its optimum enzyme activity at a higher temperature compared to the mesophilic microbes (Moguel et al. 2022). Such enzymatic behavior is not surprising since most cold-adapted enzymes have an optimal temperature higher than their physiological temperature, a function acquired during the evolutionary pressure (Bjelic et al. 2008). Various other L-ASNases are reported to exhibit maximum activity at higher temperatures viz., 60 ℃ for *Yersinia pseudotuberculosis* (Pokrovskaya et al. 2012a) and *Cobetia amphilecti* AMI6 (Farahat et al. 2020) and 65 ℃ for *Bacillus amyloliquefaciens* MKSE (Yim and Kim 2019).

Thermal stability is also important because it imparts long-term storage stability and improves protein shelf-life. Thermal stability of PCH199 L-ASNase was better than L-ASNase of *E. coli*. The latter retains 71% activity in 60 min at 50 ℃, which is further improved to 90% via mutation (Li et al. 2007). The stability of L-ASNase from other sources are comparatively lower. *Paenibacillus barengoltzii* (Shi et al. 2017), *Bacillus aryabhattai* ITBHU02 (Singh et al. 2013), halo-thermotolerant *Bacillus* strain (Safary et al. 2019), *Vibrio cholerae* (Radha et al. 2018), *Enterobacter cloacae* (Husain et al. 2016) have less stability at 50 and 37 ℃ as compared to PCH199 L-ASNase. The ability of the enzyme from PCH199 to be stable at 37 ℃ for an extended period and functional activity at a lower temperature indicated that the enzyme would be highly efficient at normal human body temperature. Additionally, the above features of PCH199 L-ASNase impart cost-effectiveness in terms of storage and transportation. The thermostability of L-ASNase in the present study is not surprising and is consistent with adaptive features acquired by psychrophilic enzymes. There is enough evidence from other studies (Cavicchioli et al. 2011; Santiago et al. 2016) and our findings (Kumar et al. 2022a; Patial et al. 2022) that cold-active enzymes are catalytic efficient, have high substrate affinity and are stable at various pH and temperatures. Fluctuating physico-chemical conditions of the cold environment bestowed the proteins with high flexibility in protein structures (Siddiqui and Cavicchioli 2006) due to decreased number and strength of various interactions (Goldstein 2007).

Metal ions such as Cu^2+^ and Co^2+^ drastically inhibited the enzyme activity in the current study. Results are similar to reports of a decrease in L-ASNase activity of *Bacillus aryabhattai* ITBHU02 (Singh et al. 2013), *Bacillus megaterium* H-1 (Zhang et al. 2015), *Paenibaeillus barengoltzii* (Shi et al. 2017) by divalent ions such as Cu^2+^ and Co^2+^. The enzyme activity inhibition by divalent ions may be due to the chelation of sulfhydryl groups of L-ASNase with metal ions (Kumar et al. 2022a). Na^+^ enhanced the activity of L-ASNase, similar to reports of *Bacillus megaterium* H-1 (Zhang et al. 2015) and *Bacillus megaterium* strain MG1 (Roy et al. 2019). The protein modifiers such as PMSF and EDTA did not affect the activity, consistent with reports for L-ASNase of *Bacillus megaterium* H-1 (Zhang et al. 2015), *Bacillus megaterium* strain MG1 (Roy et al. 2019), where activity is non-significantly affected by EDTA and PMSF. The above outcome is well supported by the fact that L-ASNase from strain PCH199 lacks serine in it. Besides, the effect of SDS was negligible and DMSO moderately decreased the activity of the purified enzyme, comparable to *Bacillus megaterium* strain MG1 (Roy et al. 2019) with 77% decreased activity in presence of DMSO.

L-asparagine concentration in human blood is approximately 50 μM. Therefore, potential therapeutic L-ASNases must have higher substrate affinity (Ollenschläger et al. 1988; Nguyen et al. 2016). The lower* K*_m_ value of 0.164 mM of the enzyme indicated high substrate-specificity, which is vital in therapeutic applications. Various studies have revealed that L-ASNase from different microbial sources varies in its affinity towards L-asparagine (Kishore et al. 2015). *Pseudomonas oryzihabitans* exhibit higher *K*_m_ of 10 mM than PCH199 (Bhagat et al. 2016). Similarly, *Bacillus altitudinis* exhibited a higher *K*_m_ value of 90.9 mM (Prakash et al. 2020). A few bacteria reported to have higher *K*_m_ values are *Bacillus halotolerans* (4.7 mM) (El-Fakharany et al. 2022), *Bacillus amyloliquefaciens* (1.5 mM) (Yim and Kim 2019) and *Paenibacillus barengoltzii* (3.6 mM) (Shi et al. 2017). Even though the *K*_m_ of PCH199 is higher than the commercially existing *E. coli* (14.9 µM) and *Dickeya* L-ASNase (47.5 µM) (Schalk et al. 2014), it is still better compared to the various microbial L-ASNases reported. Biochemical and kinetic analysis revealed PCH199 L-ASNase to be a highly stable enzyme at 37 and 50 ℃, broad pH and temperature activity in addition to comparable *K*_m_ value for L-asparagine. Such parameters could be useful in the pharmaceutical and food industries.

The anticancer efficacy of PCH199 L-ASNase indicated the effective killing of leukemic cell lines because the deamination of non-essential amino acid asparagine results in a depleted asparagine pool (Saeed et al. 2020). There are reports in the literature supporting the effectiveness of L-ASNase against blood cancer cell lines i.e., *Melioribacter roseus* (IC_50_ 3.0 U/mL) (Dumina et al. 2021), *Rhodospirillum rubrum* (IC_50_ 1.80 U/mL) (Pokrovskaya et al. 2012b), and *Halomonas elongate* (IC_50_ 2.0 U/mL; 1.0 U/mL) (Ghasemi et al. 2017). No cytotoxic effect was observed for normal cell lines. Instead, proliferation was observed. This can be attributed to the activity of asparagine synthetase that uses substrate provided by other processes during depletion. The lower the IC_50_ value, the more efficacious the drug is at low doses, and consequently, lower the systemic toxicity when administered to patients (Berrouet et al. 2020). The IC_50_ value of PCH199 is comparatively very low, indicating a potential drug with a lower dosage.

Apoptosis is a critical mechanism used by various chemotherapeutic agents as their anti-proliferative effect (Lowe and Lin 2000). Reports of L-ASNase from *Enterobacter cloacae* (Husain et al. 2016) and *Zymomonas mobilis* (Einsfeldt et al. 2016) show the nuclear morphological changes induced by L-ASNase. Thus, as observed using the DAPI staining method, purified PCH199 L-ASNase causes apoptotic cell death indicated by nuclear morphological changes in human leukemic cells. With low *K*_m_ and IC_50_ values of 0.164 mM and 0.309 U/mL, respectively, the enzyme’s ability to induce apoptosis along with high stability at 37 ℃ (human physiological temperature) and 50 ℃, makes it a valuable bioproduct in the pharmaceutical and food industries.

In conclusion, the study revealed a Himalayan bacterium *Pseudomonas* sp. PCH199 with type II periplasmic L-ASNase activity. Statistical optimization revealed the importance of buffer and L-asparagine to enhance enzyme production. Physicochemical parameters revealed the functional stability of the enzyme in a wide range of pH and temperature. These properties can be beneficial in the therapeutic and food industries. Even though the L-GLNase activity related side effects are debated, the PCH199 L-ASNase also exhibited L-GLNase activity. Nevertheless, the unique feature such as a high degree of stability in human physiological conditions, high substrate-specificity, and efficient cytotoxicity against K562 blood cancer cell lines, PCH199 L-ASNase have displayed efficient and robust attributes for potential applications in therapeutics. In-vivo cytotoxicity validation and its efficacy determination in addition to deletion of L-GLNase activity by mutagenesis, take it a step further to establish PCH199 as a drug for ALL.

## Supplementary Information

**Additional file 1: Fig. S1 **Qualitative estimation of L-ASNase production by PCH199. The strain *Pseudomonas *sp. PCH199 was streaked on M9 medium supplemented with indicator dye phenol red. After 24h of incubation at 28 ℃, color change around the bacterial colony from yellow to pink has indicated L-asparagine hydrolysis by L-ASNase due to change in pH. **Fig. S2 **Phylogenetic tree of *Pseudomonas *sp. PCH199 with related strains based on 16S rRNA gene sequence analysis. The tree was constructed by maximum likelihood method using MEGA11 with 1000 boot-strapping replication. PCH199 showed 99.06 % sequence similarity to *Pseudomonas glycinae *MS586(T). **Fig. S3 **Graphical representation of kinetic study of PCH199 L-ASNase. Determination of *K*m and *V*max of purified L-ASNase for L-glutamine by non-linear regression analysis of experimental steady-state data. (a) Plot of the reaction velocities (V) versus substrate concentration (S: 0.02 – 0.5 mM) fitted to the Michaelis-Menten equation. (b) The corresponding Lineweaver-Burk plot (*K*m = 0.034 mM and *V*max = 57.98 U/mg) of L-ASNase catalyzed reaction. **Fig.S4 **Graphical representation of periplasmic L-ASNase activity when PCH199 was cultured at M9 minimal medium with various pH (5.8-7.5) and periplasmic L-ASNase activity corresponding to each pH was represented. **Table S1.** Central Composite Design of selected variables and the responses thereof. The maximum response obtained with a specific condition is marked in bold font. **Table S2.** Analysis of variance of second-order polynomial model for the effect of different variables on L-ASNase production.

## Data Availability

The 16S rRNA gene sequence of the strain PCH199 has been submitted to the NCBI GenBank with accession number ON782287. The culture is submitted to the patent deposit of Microbial Type Culture Collection (MTCC) with accession number 25172.

## Abbreviations

ALL: Acute lymphoblastic leukemia
TCA: Trichloroacetic acid
RSM: Response surface methadology
CCD: Central composite design
MTT: [3-(4, 5-Dimethylthiazol-2-yl)-2,5-diphenyl tetrazolium bromide
DAPI: 4′,6-Diamidino-2-phenylindole dihydrochloride
IC_50_: Half maximal inhibitory concentration
