# Supplementary material for: Biochemical characterization of extremozyme L-asparaginase from Pseudomonas sp. PCH199 for therapeutics
Source: AMB Express. 2023 Feb 24;13:22. doi: 10.1186/s13568-023-01521-2 (PMC9958223; doi:10.1186/s13568-023-01521-2)
Supplement: Supplementary file 1 — Additional file 1: Fig. S1 Qualitative estimation of L-ASNase production by PCH199. The strain Pseudomonas sp. PCH199 was streaked on M9 medium supplemented with indicator dye phenol red. After 24h of incubation at 28 ℃, color change around the bacterial colony from yellow to pink has indicated L-asparagine hydrolysis by L-ASNase due to change in pH. Fig. S2 Phylogenetic tree of Pseudomonas sp. PCH199 with related strains based on 16S rRNA gene sequence analysis. The tree was constructed by maximum likelihood method using MEGA11 with 1000 boot-strapping replication. PCH199 showed 99.06 % sequence similarity to Pseudomonas glycinae MS586(T). Fig. S3 Graphical representation of kinetic study of PCH199 L-ASNase. Determination of Km and Vmax of purified L-ASNase for L-glutamine by non-linear regression analysis of experimental steady-state data. (a) Plot of the reaction velocities (V) versus substrate concentration (S: 0.02 – 0.5 mM) fitted to the Michaelis-Menten equation. (b) The corresponding Lineweaver-Burk plot (Km = 0.034 mM and Vmax = 57.98 U/mg) of L-ASNase catalyzed reaction. Fig.S4 Graphical representation of periplasmic L-ASNase activity when PCH199 was cultured at M9 minimal medium with various pH (5.8-7.5) and periplasmic L-ASNase activity corresponding to each pH was represented. Table S1. Central Composite Design of selected variables and the responses thereof. The maximum response obtained with a specific condition is marked in bold font. Table S2. Analysis of variance of second-order polynomial model for the effect of different variables on L-ASNase production. [file 13568_2023_1521_MOESM1_ESM.pdf]

Title: Biochemical characterization of extremozyme L-asparaginase from *Pseudomonas* sp. PCH199 for therapeutics

Authors: Sanyukta Darnal[1,2], Vijeta Patial[1,2], Virender Kumar[1], Subhash Kumar[1,2], Vijay Kumar[1], Yogendra S. Padwad[2,3], Dharam Singh[1,2*]

Authors Affiliations:

[1]CSIR-Institute of Himalayan Bioresource Technology, Palampur, Himachal Pradesh-176 061, INDIA.

[2]Academy of Scientific and Innovative Research (AcSIR), Ghaziabad 201 002, INDIA.

[3]Dietetics & Nutrition Technology Division, CSIR-Institute of Himalayan Bioresource Technology, Palampur, Himachal Pradesh-176 061, INDIA

***Corresponding Author**

Molecular and Microbial Genetics Lab, Biotechnology Division,

CSIR-Institute of Himalayan Bioresource Technology,

Palampur, Himachal Pradesh, INDIA-176 061

Phone: +91-1894-233339, ext. 422

Fax: + 91-1894-230433

E-mail: dharamsingh@ihbt.res.in; dharams14@gmail.com

Fig. S1 Qualitative estimation of L-ASNase production by PCH199. The strain *Pseudomonas* sp. PCH199 was streaked on M9 medium supplemented with indicator dye phenol red. After 24h of incubation at 28 °C, color change around the bacterial colony from yellow to pink has indicated L-asparagine hydrolysis by L-ASNase due to change in pH.

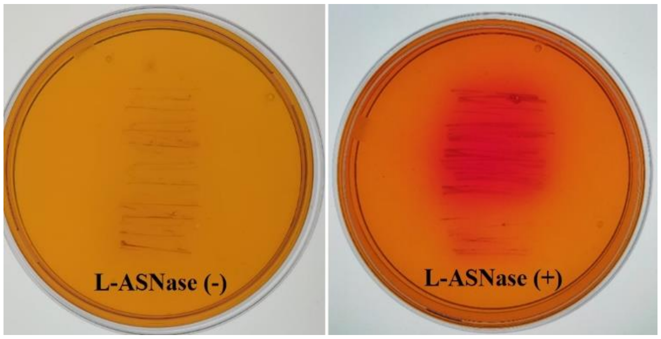

Fig. S2 Phylogenetic tree of *Pseudomonas* sp. PCH199 with related strains based on 16S rRNA gene sequence analysis. The tree was constructed by maximum likelihood method using MEGA11 with 1000 boot-strapping replication. PCH199 showed 99.06 % sequence similarity to *Pseudomonas glycinae* MS586(T).

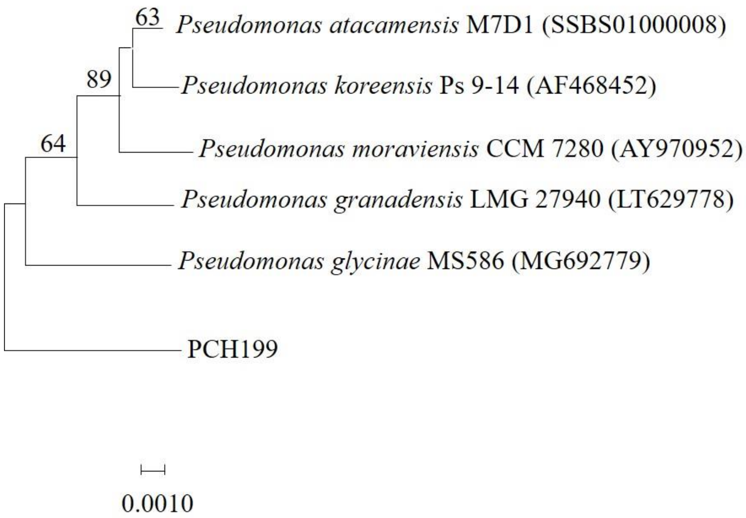

Fig. S3 Graphical representation of kinetic study of PCH199 L-ASNase. Determination of K_m and V_{max} of purified L-ASNase for L-glutamine by non-linear regression analysis of experimental steady-state data. (a) Plot of the reaction velocities (V) versus substrate concentration (S: 0.02 – 0.5 mM) fitted to the Michaelis-Menten equation. (b) The corresponding Lineweaver-Burk plot (K_m = 0.034 mM and V_{max} = 57.98 U/mg) of L-ASNase catalyzed reaction.

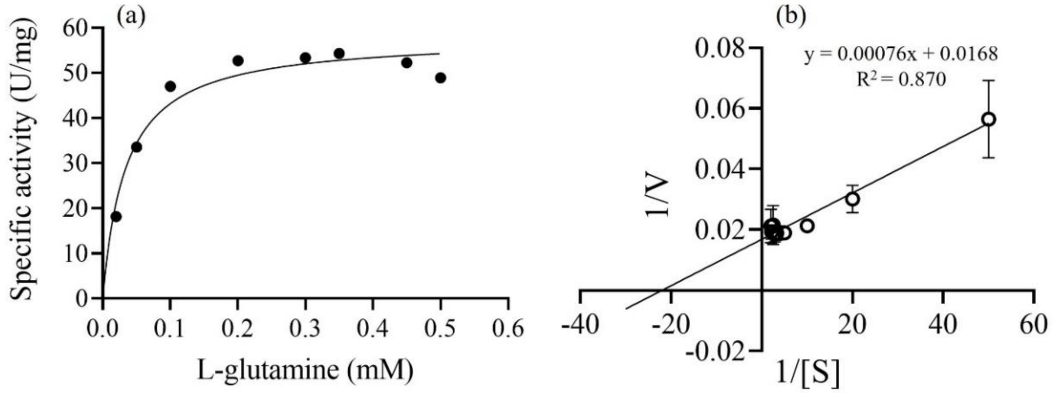

Fig.S4 Graphical representation of periplasmic L-ASNase activity when PCH199 was cultured at M9 minimal medium with various pH (5.8-7.5) and periplasmic L-ASNase activity corresponding to each pH was represented.

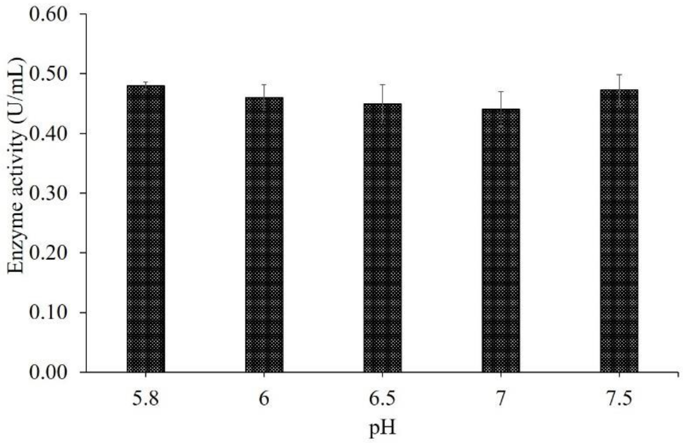

Table S1 Central Composite Design of selected variables and the responses thereof. The maximum response obtained with a specific condition is marked in bold font.

Run Order	Buffer concentration (mM)	L-asparagine concentration (%)	Glucose concentration (%)	Response (U/mL)
1	50	2.732	0.25	0.541
2	50	2.732	0.25	0.664
**3**	**100**	**2.0**	**0.5**	**0.766**
4	0	2.0	0	0.038
5	0	2.0	0.5	0.009
6	100	2.0	0	0.635
7	50	1.0	0.25	0.456
8	50	1.0	0.25	0.444
9	50	1.0	-0.183	0.328
10	-36.602	1.0	0.25	0.232
11	50	1.0	0.683	0.437
12	136.603	1.0	0.25	0.553
13	50	1.0	0.25	0.456
14	50	1.0	0.25	0.451
15	50	-0.732	0.25	0.048
16	50	-0.732	0.25	0.036
17	100	0	0.5	0.026
18	0	0	0.5	-0.024
19	0	0	0	-0.019
20	100	0	0	0.046

Table S2 Analysis of variance of second-order polynomial model for the effect of different variables on L-ASNase production.

Source	Term df	Error df	F-value	p-value	Remark
Whole-plot	2	3.46	9.51	0.0394	Significant
b-Asparagine concentration	1	3.38	16.97	0.0205	
b^2	1	3.53	2.05	0.2347	
Subplot	7	6.55	14.40	0.0016	Significant

A-Salt concentration	1	7.20	56.62	0.0001
C-Glucose concentration	1	7.20	1.45	0.2662
Ab	1	7.20	42.13	0.0003
AC	1	7.20	0.9731	0.3559
bC	1	7.20	0.1425	0.7167
A^2	1	3.83	2.28	0.2089
C^2	1	3.83	2.52	0.1906
R^2 (predicted) = 0.954				
R^2 (adjusted) = 0.892				

df, indicates for degree of freedom.
